# Radio Wave-Activated Chemotherapy—A Novel Nanoparticle Thermoresponsive Copolymer Drug Delivery Platform

**DOI:** 10.3390/ma16062482

**Published:** 2023-03-21

**Authors:** Benjamin D. White, Helen E. Townley

**Affiliations:** 1 Department of Engineering Science, University of Oxford, Parks Road, Oxford OX1 3PJ, UK; 2 Department of Women’s and Reproductive Health, John Radcliffe Hospital, University of Oxford, Oxford OX1 3PJ, UK

**Keywords:** cancer, nanoparticles, chemotherapy, radio waves, activation, responsive, smart

## Abstract

Radio waves are highly penetrating, non-ionizing, and cause minimal damage to surrounding tissues. Radio wave control of drug release has been achieved using a novel thermoresponsive copolymer bound to a superparamagnetic iron oxide nanoparticle (SPION) core. A NIPAM-acrylamide-methacrolein copolymer underwent a coil-to-globular structure phase change upon reaching a critical temperature above the human body temperature but below hyperthermic temperatures. The copolymer was covalently bound to SPIONs which increase in temperature upon exposure to radio waves. This effect could be controlled by varying input energies and frequencies. For controlled drug release, proteins were bound via aldehyde groups on the copolymer and amine groups on the protein. The radio wave-induced heating of the complex thereby released the drug-bearing proteins. The fine-tuning of the radio wave exposure allowed multiple cycles of protein-drug release. The fluorescent tagging of the complex by FITC was also achieved in situ, allowing the tagging of the complex. The localization of the complex could also be achieved in vitro under a permanent magnetic field.

## 1. Introduction

To improve the therapeutic index of chemotherapy treatments, greater control over the site of action is needed. This can be partially achieved through the use of nanoparticle delivery systems; however, further refinement can be attained by controlled release. Nanoparticle delivery systems are an ideal size for extravasation from the vascular system into tissues and movement across cell membranes into the cytoplasm. In addition to passive targeting by the enhanced permeation and retention (EPR) effect, active targeting can be achieved by the addition of antibodies or aptamers which recognize surface markers on specific cells. Drug circulation times may also be greatly increased by attachment to nanoparticles or encapsulation, improving the drug half-life [1]. The size, shape, and charge of nanoparticles will also affect circulation times and uptake rates by cells [2,3].

Controlled release may be achieved via intrinsic or extrinsic mechanisms. Intrinsic release mechanisms rely on changes in the cell chemistry as a consequence of altered cancer cell metabolism. Extrinsic activation permits controlled release from a source outside of the cell. Herein, we exploit radio waves (alternating magnetic field; AMF) for extrinsic activation due to their inert nature and high penetration depth. Radio waves can cause inductive heating in single-crystal superparamagnetic magnetite (Fe_3_O_4_) nanoparticles [4]. Such superparamagnetism occurs when the Neel relaxation of a magnetic particle is shorter than the measurement time of its magnetic domain [5,6,7]. At low and medium radio frequencies (100–800 kHz), superparamagnetic iron oxide nanoparticles (SPIONs) absorb electromagnetic energy as thermal energy while in solution [7,8,9]. This effect is explained by the dissipation relationships of rotational relaxation of single-domain magnetic particles dispersed in a liquid medium [10]. The extent of heating is dictated by a number of factors including the intensity of radiation, the environment of particles, size, and the morphology of particles [11]. Since the majority of tissues in the body have no absorption in the radio wave spectrum, there is little possibility of off-target effects as seen with shorter wavelengths in treatment such as radiotherapy [12].

Although this heating effect can be exploited for hyperthermia, this is often uncontrolled and leads to off-target cell death. However, this phenomenon can be used in conjunction with novel thermosensitive polymers for greater control. A commonly studied example is poly-N-isopropylacrylamide (PNIPAM) which is biocompatible and water soluble [13]. While in an aqueous solution at a low temperature, PNIPAM adopts a coil-like structure to maximize the enthalpy of hydration through hydrogen bonding between the amide group within the polymer and the surrounding water molecules. A temperature-induced phase transition involves the coil structure of PNIPAM collapsing into a hydrophobic globular structure, releasing water molecules [14]. Upon heating above 32 °C, the polymer undergoes a reversible lower critical solution phase transition temperature (LCST). Crucially, this would not be suitable for in vivo applications since the human body temperature is 37 °C [15]. In order to alter the transition temperature, PNIPAM can be copolymerized with hydrophilic monomers such as acrylamide, N-acryloyl morpholine, and dimethyl acrylamide [16,17,18,19,20]. In the current study, acrylamide was used due to its availability, easy manipulation, and reactivity ratio with NIPAM [21]. A third monomer, methacrolein, was also incorporated since it is capable of radical polymerization via its alkene group and also possesses an aldehyde functional group to enable binding to proteins [22].

Proteins have previously been used as carrier moiety for chemotherapeutic agents. Herein, bovine serum albumin (BSA) was used for proof of concept and bound to the common chemotherapy drugs, paclitaxel and vinblastine. Consequently, the complex comprises a SPION core, a covalently bound thermosensitive copolymer, and drug-bearing proteins. The application of radio waves causes inductive heating in the SPION, increasing the temperature of the solution, triggering the phase change of the copolymer, and releasing drug-bearing proteins. Furthermore, spatial localization can be achieved by implementing permanent magnetic fields. Therefore, it has been shown for the first time that an N-isopropylacrylamide–acrylamide–methacrolein copolymer is capable of immobilizing drug-bearing proteins and undergoing a phase transition at a desired temperature of 40–42 °C. The resulting thermoresponsive core–shell nanoparticles combine the advantages of remote activation and release with non-ionizing radiation.

## 2. Materials and Methods

### 2.1. Iron Oxide Nanoparticle Poly-(methacrolein-b-(NIPAM-co-acrylamide) Synthesis

The magnetite SPION core of the nanoparticle–copolymer complex (NCC) was synthesized in a coprecipitation reaction. This was conducted by dissolving 0.56 g of iron (II) chloride and 2.35 g of iron (III) chloride hexaqua in 25 mL of ddH_2_O under a nitrogen atmosphere. The solution was gradually heated to 80 °C, and during heating, 0.1 g of lauric acid dissolved in 5 mL acetone was added. This was immediately followed with the addition of 5 mL of pre-mixed 28% (*v/v*) ammonium hydroxide. Once the solution had reached 80 °C, 5 aliquots of 0.2 g of lauric acid in 5 mL acetone were added over 5 min. The solution was allowed to cool and precipitated out in 100 mL ethanol:acetone (50:50 *v/v*; all Sigma-Aldrich Merck Life Science UK Limited, Gillingham, UK), and the product was removed by magnetic decantation.

To enable polymerization on the surface of the SPION core, the stabilizing ligand lauric acid was exchanged with the RAFT chain transfer agent 2-(Dodecylthiocarbonothioylthio)-2-methylpropionic acid (DDMAT).

To enable ligand exchange, 200 mg of freshly synthesized lauric acid-stabilized iron oxide nanoparticles was dispersed in 20 mL of 1,2-dichlorobenzene. DDMAT (1 g) was then added, and the solution was stirred at 80 °C for 24 h. The DDMAT-stabilized nanoparticles (DD-ION) were precipitated in methanol, extracted with a magnet, and washed in several cycles of dispersion in tetrahydrofuran and re-precipitation in methanol (all materials: Sigma-Aldrich Merck Life Science UK Limited, Gillingham, UK).

Copolymer synthesis was performed on the surface of the DD-ION particles, with polymer chains binding to the chain transfer agent DDMAT present on the surface of the SPION cores. DD-ION (100 mg) was dissolved in 5 mL dimethyl fumarate under a nitrogen atmosphere. Methacrolein (79 μL, 1 mmol) was added to the solution followed by 1.17 mg Azobisisobutyronitrile (AIBN) which acted as the initiator, still under a nitrogen atmosphere. The reaction flask was then degassed by three freeze–thaw pump cycles and heated to 65 °C and stirred for 2 weeks. The long reaction time was necessary due to the low reactivity of the methacrolein monomer. Following this, N-isopropyl acrylamide (4.5 mmol, 508 mg) and acrylamide (4.5 mmol, 380 mg) were added to the reaction mixture, still under a nitrogen atmosphere. After a further 48 h, the solution was diluted with 35 mL tetrahydrofuran, precipitated in 40 mL diethyl ether (all Sigma-Aldrich Merck Life Science UK Limited, Gillingham, UK), and removed by magnetic decantation; this was repeated three times.

Unbound polymers (not bound to SPION) were synthesized as above with 25 mg of DDMAT used in place of DD-ION.

### 2.2. Drug-Bearing Protein Immobilization

Drug binding to BSA (Sigma-Aldrich, Gillingham, UK) was performed before immobilization on the nanoparticle–polymer complex to ensure efficient uptake. To begin, 1.2 mg paclitaxel (Sigma-Aldrich, Gillingham, UK) was dissolved in 100 μL pure ethanol. This was combined with 9.9 mL pure H_2_O containing 6 mg BSA, and the solution was stirred at 21 °C for 3 h. Vinblastine (Sigma-Aldrich, Gillingham, UK) attachment was performed akin to paclitaxel, with the equivalent molar ratio of vinblastine (1.27 mg) in place of paclitaxel. The BSA–drug complex was removed by precipitation with 10 mL ethanol and centrifugation at 10,000× *g* for 5 min. The precipitate was then washed three times with ethanol and lyophilized. The paclitaxel content was measured by the complete degradation of the BSA using trypsin incubation for 30 min at 37.5 °C. The released paclitaxel was quantified by HPLC measurement and peak integration. For the drug detection of chemotherapy agents, samples were prepared at a concentration of 0.2–0.3 mg mL^−1^ in PBS. Samples of paclitaxel were analyzed on an Agilent 1120 compact LC. The column used for the stationary phase was a ZORBAX Eclipse 18C β-Carotene column (4.6 × 150 mm, 5 microns), and detection was run at 228 nm with a deuterium lamp. The mobile phase for the separation of paclitaxel consisted of 58 % (*v/v*) acetonitrile and 42 % (*v/v*) HPLC-grade water (all Sigma-Aldrich, Gillingham, UK). A paclitaxel peak was detected at 5.88 min and each run had a finishing time of 6 min. Vinblastine was measured using the same method but with the detection wavelength set to 245 nm, and the run length time was 5 min.

The penultimate step of NCC–drug synthesis was as follows: An equilibrium solution was formed with 8 mg of the free copolymer or 16 mg of the nanoparticle–copolymer complex, and 6 mg of BSA–Drug complex in 5 mL PBS. The solution was cooled to 4 °C and stirred for 24 h.

Finally, 50 mg of fluorescein isothiocyanate (FITC; Sigma-Aldrich, Gillingham, UK) was added, and the solution was stirred at 4 °C for another 24 h. Following this, the particles were precipitated in ethanol and extracted by magnetic decantation before redispersion in water. This wash step was repeated three times before the particles were lyophilized.

### 2.3. Inductive Heating

Inductive heating was carried out using a 2000W ZVS low voltage induction heating board with a module Tesla coil and a flyback driver heater. The working coil consisted of a copper tube wound with 10 coils and a 6-inch coil diameter. The coil was water-cooled to prevent external heat input to the sample. The coil had a sufficient diameter to allow cell culture plates to fit inside the coil. A variable laboratory power supply system was used to supply the circuit board with current and voltage. To assess the heating times in the cell culture, preliminary tests were run in non-sterile conditions with particles dispersed in PBS. The voltage was varied by the power supply, and the temperature of the solution was measured every minute using a non-mercury or non-conductive thermometer. For the activation of nanoparticles in the cell culture, the plate was placed inside the inductor coil and insulated. Once the desired temperature had been reached, the plate was removed and returned to the incubator. Input voltages and heating times were dependent upon the concentration of particles used as measured in non-sterile conditions.

### 2.4. Analysis Methods

The zeta potential of the nanoparticles was measured using a Malvern Instruments Zetasizer Nano (Microtrac UK, Verder Scientific UK Ltd., Hope Valley, UK). For the drug detection of chemotherapy agents, samples were prepared at a concentration of 0.2–0.3 mg mL^−1^ in PBS. Samples of paclitaxel were analyzed on an Agilent 1120 compact LC (Agilent technologies, 5301 Stevens Creek Blvd. Santa Clara, CA, USA). The column used for the stationary phase was a ZORBAX Eclipse C18 column (4.6 × 150 mm, 5 microns), and detection was run at 228 nm with a deuterium lamp. The mobile phase for the separation of paclitaxel consisted of 58% (*v/v*) acetonitrile and 42% (*v/v*) HPLC-grade water. A paclitaxel peak was detected at 5.88 min and each run had a finishing time of 6 min. Vinblastine was measured using the same method but with the detection wavelength set to 245 nm, and the run length time was 5 min. TEM images were taken using a JEOL JEM-2100 Plus microscope (JEOL House, Welwyn Garden City, UK). Samples (5 mg) were dissolved in ethanol or tetrahydrofuran depending on solubility before dispersion on holey carbon grids (Sigma-Aldrich, Gillingham, UK). The samples were then imaged under a 200 KV beam.

For GPC analysis samples were prepared at concentrations of 2 mg/mL in DMF with 5 mM NH_4_BF_4_. Samples were run on an Agilent Infinity II MDS instrument equipped with a differential refractive index (DRI), viscometry (VS), dual-angle light scatter (LS), and variable wavelength UV detectors. The system was equipped with 2 × PLgel Mixed D columns (300 × 7.5 mm) and a PLgel 5 µm guard column. Samples were run at 1 mL/min at 50 °C. Poly(methyl methacrylate) standards (Agilent EasiVials, Santa Clara, CA, USA) were used for calibration between 955,000–550 gmol^−1^. Analyte samples were filtered through a nylon membrane with 0.22 μm pore size before injection. Respectively, experimental molar mass (Mn, SEC) and dispersity (Đ) values of synthesized polymers were determined by conventional calibration and universal calibration using Agilent GPC/SEC software. The GPC chromatography was run on polymers synthesized in free solution and polymers synthesized on the surface of SPIONs. The polymers synthesized on SPIONs were removed for analysis by a ligand exchange reaction.

Fluorescence spectroscopy was performed on a Cary Eclipse fluorescence spectrometer using Ex. 495 nm and Em. 500–700 nm (2.5 nm slits) at 25 °C (Agilent Technologies, 5301 Stevens Creek Blvd., Santa Clara, CA, USA). Fluorescence imaging was performed on a Versa doc molecular imager (Molecular Imager 4000 system, VersaDoc™ MP Imaging Systems, Watford, UK) using Ex. 495 nm and Em. 520 nm.

PerkinElmer Frontier IR/FTIR (Seer Green, Beaconsfield, UK) was used for the determination of surface functional groups on a sample, with a scan range from 600–4000 cm^−1^. Multinuclear NMR was assessed for both C^13^ and H^1^ using a 500 MHz Bruker Avance (Bruker Instruments, Coventry, UK). The free polymers and polymers exchanged from grafting on nanoparticles were dispersed in deuterium oxide. MestReNova version 11.0.01 software was used to analyze the spectra.

### 2.5. Cell Culture and Viability Experiments

Immortalized cancer cell lines were obtained from the American Type Culture Collection (ATCC; Manassas, VA, USA). The two cell lines tested were RD (ATCC code CCL-136) and U-87 MG (ATCC code HTB-14). The cells were maintained in a growth medium (Dulbecco’s modified Eagle’s medium; Sigma-Aldrich, Poole, UK) supplemented with 10% (*v/v*) fetal calf serum, 2 mM L-glutamine, 100 U/mL penicillin, and 0.1 mg/mL streptomycin (all Sigma-Aldrich) and then incubated at 37 °C in a 5% CO_2_ atmosphere. The cells were passaged every 3–4 days.

The cells were seeded in 96 well plates at 1 × 10^4^ cells/well in growth media and left overnight in the incubator for the cells to adhere. The cells were then treated with different concentrations of the nanoparticle–copolymer complex and controls in a 50 μL growth medium. After treatment, the media on the cells were removed and the cells were washed in PBS (Sigma-Aldrich Merck Life Science UK Limited, Gillingham, UK) twice. The experiments were performed in triplicates and repeated on three separate occasions.

### 2.6. Flow Cytometry Experimental Procedure

The cells were seeded in 24 well plates at 8 × 10^4^ cells/well in growth media and left overnight in the incubator for the cells to adhere. The cells were then treated with different concentrations of NCC and controls in a 50 μL growth medium. After treatment, the media on the cells were removed and the cells were washed in PBS twice. The harvested cells were aliquoted up to 1 × 10^6^ cells/100 μL into FACS tubes (Merck KGaA, Darmstadt, Germany). The cells were centrifuged in 2 mL PBS at 300× *g* for 5 min, and then the buffer was decanted from the pelleted cells. This process was repeated a total of 3 times. To adjust flow cytometer settings for propidium iodide (PI; Sigma-Aldrich, Gillingham, UK). PI staining solution (5–10 μL) was added to a control tube of otherwise-unstained cells. The cells were mixed gently and incubated for 15 min in the dark. PI fluorescence was determined (using the FL-3 channel) with a BD Sciences FACSCalibur™ Flow Cytometer (1 Becton Drive Franklin Lakes, NJ, USA). Collected PI fluorescence was gathered in the FL-3 channel. To each sample, 5–10 µL of PI staining solution was added 15 min prior to analysis. The stop count for events was set at 10,000.

### 2.7. Spheroid Preparation

Spheroids were created in 96-well plates. Agarose (1% (*w/v*); (Munro House, Trafalgar Way, Bar Hill, Cambridge, UK) was dissolved in PBS and heated in the microwave for 6 min, before the addition of 100 μL to each well in a 96-well plate. The gel was allowed to cool for 1 h undisturbed so that a non-adherent, concave surface was formed. While cooling, UV light was used for the decontamination of the agarose surface. The cells were then seeded (1 × 10^4^ per well) in 100 μL cell growth medium (Dulbecco’s modified Eagle’s medium [Sigma-Aldrich]) supplemented with 10% fetal calf serum, 2 mM L-glutamine, 100 U/mL penicillin, and 0.1 mg/mL streptomycin (all Sigma-Aldrich, Gillingham, UK). The plates were then incubated at 37 °C in a 5% CO_2_ atmosphere for 3 to 4 days to allow the formation of spheroids.

### 2.8. Haemolytic Activity of ION-Polymer Complex

Fresh horse blood stabilized with heparin (Generon Clinical Sciences, Slough, UK) was used to assess the hemolytic activity of the nanoparticle–polymer complex. Healthy red blood cells (HRBCs) were isolated from 1 mL of fresh horse blood by centrifugation at 1000 rpm for 10 min at 4 °C and washed with PBS at least three times. HRBCs were then diluted ten times with PBS buffer. The diluted HRBC suspension (0.1 mL) was added to 1 mL of water (positive control), pure PBS (negative control), or PBS buffer containing the nanoparticle–polymer complex ranging from 0.1 to 1 mg/mL. After gentle shaking, the mixtures were kept still for 24 h at room temperature in different batches with triplicates. Then, after the centrifugation of the mixture (5000 rpm, 1 min), the absorbance of the supernatant (hemoglobin) was recorded using a Perkin Elmer Lambda 25 UV–VIS spectrometer (PerkinElmer, Inc., 940 Winter Street, Waltham, MA, USA). The extent of hemolysis was calculated by dividing the difference in absorbance between the sample and the negative control by the difference in absorbance at 541 nm between the positive and negative controls.

### 2.9. Magnetic Directing Capacity of ION–Polymer Complex

Two separate plates of cells were set up for the magnetic direction and fluorescence treatment of cells and the other for control cells. The cells were seeded in 6-well plates at 20 × 10^4^ cells/well in 2.5 mL growth media and left overnight in the incubator at 37.5 °C (5% CO_2_) for the cells to adhere. The cells were then treated with 0.2 mg/mL of FITC-NCC for 24 h. After treatment, the media from both plates of cells were removed, and the cells were washed in PBS three times before harvesting with 200 μL of trypsin (Sigma-Aldrich Merck Life Science UK Limited, Gillingham, UK). The cells incubated with NCC and the control cells were merged in a third six-well plate in a 2.5 mL growth medium in the presence of a permanent magnetic field and allowed to settle for 24 h. Subsequently, the plates were imaged under fluorescence.

## 3. Results and Discussion

### 3.1. Thermosensitive Copolymer Design Synthesis and Analysis

**Figure 1:**
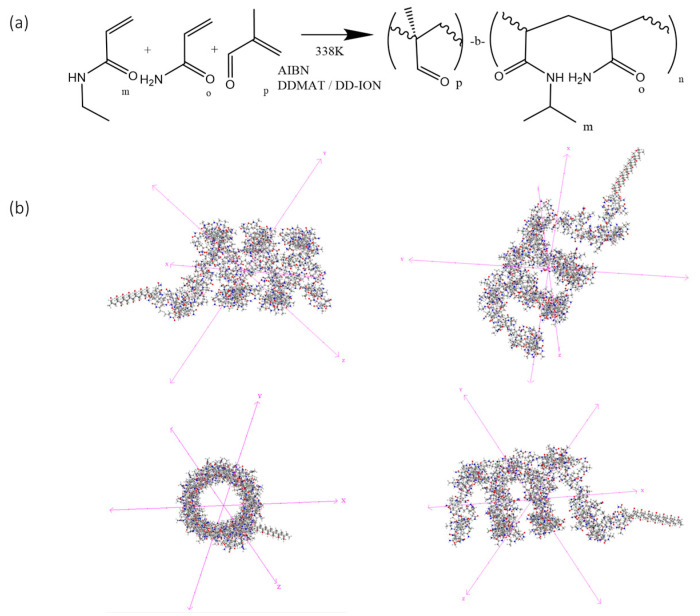
Polymerization synthesis. (**a**) Process and resulting block structure of the novel copolymer NIPAM–acrylamide–methacrolein. (**b**) The three-dimensional structure of the synthesized copolymer showing distinct block copolymer regions as viewed along different axes. The methacrolein block and the NIPAM–acrylamide coil structure are visible as two distinct regions.

Currently used copolymers have an LCST below human body temperature. To achieve a transition temperature in the range of 41–43 °C, a novel thermosensitive copolymer was designed. The copolymer was designed with three components; (i) NIPAM as the backbone of the polymer to provide an LCST, (ii) acrylamide to alter the temperature at which the LCST occurs, and (iii) methacrolein as a protein-immobilizing component (Figure 1a). The NIPAM–acrylamide–methacrolein copolymer was first synthesized in a free solution. After the reaction, a yield of 57% (±5.3%) was obtained. The resulting copolymer structure was assumed to be polymethacrolein-b-(NIPAM-co-acrylamide) (Figure 1a,b). To confirm the inclusion of the component monomers, NMR, FT-IR, LCST measurements, and protein immobilization analysis were performed (Supplementary Figures S1–S4).

The FT-IR of the synthesized copolymer exhibits several main absorption features confirming the identity of the product (Supplementary Figure S2). The 3298 cm^−1^ signal corresponds to the medium N-H-stretching aliphatic primary amide of NIPAM, and hydrogen bonding broadens the peak. A signal at 3206 cm^−1^ corresponds to the medium N-H-stretching aliphatic primary amine of the acrylamide component, and again, hydrogen bonding broadens the peak. The signal present at 2969–2931 cm^−1^ corresponds to the medium two C-H-stretching alkane environments, polymer backbone, and methyl groups. The 2830–2695 cm^−1^ signal corresponds to the medium C-H-stretching aldehyde of the methacrolein component. The 1680 cm^−1^ signal corresponds to the strong C=O stretching secondary amide both acrylamide and NIPAM masking C=O of methacrolein. Lastly, the C-H bending 1390–1380 cm^−1^ signal corresponds to the methacrolein aldehyde.

To further confirm the composition of the copolymer, in situ H^1^ and C^13^ NMR (Supplementary Figure S3) was performed. The NMR data obtained from the copolymer supports the creation of a copolymer with the intended structure (Figure 1a). The signal from the C=O peak is very small due to the concentration of groups within the copolymer, and so the peak is masked by noise. When compared to the spectrum of pure PNIPAM, the presence of the peaks at δ9.5 ppm and δ181 ppm in the H^1^ and C^13^ spectra indicate the inclusion of acrylamide and methacrolein, respectively. The inclusion of several different environments between δ165 and 175 ppm suggests the presence of more than one amide molecular environment within the copolymer.

The protein immobilization of the copolymer with respect to BSA was assessed using a standard Bradford protein assay. The copolymer immobilized 9% by its own weight of BSA (Supplementary Figure S5). Further to this, it was shown to be capable of readily releasing immobilized proteins upon triggering the LCST. This suggests efficient Schiff base linkage and the effective expulsion of the drug-carrying moiety upon heating.

To achieve the desired LCST of the synthesized copolymer, the ratio of the constituent monomers NIPAM and acrylamide was varied. PNIPAM has a natural LCST of 32 °C, and due to the hydrophobicity of methacrolein, a copolymer consisting of just these two components would show a phase change of well below the target of 41–43 °C. Upon increasing the ratio of acrylamide, the LCST increased at a linear rate (Supplementary Figure S4). This observed effect is a result of the high hydrophilicity of acrylamide compared to the other monomers [22]. A higher ratio of acrylamide increased the overall hydrophilicity of the polymer, leading to a more negative value for the enthalpy of hydration and conversely a more positive value for the enthalpy of dehydration.

The phase change transition of the polymer from a coil to a globular structure must expel water molecules, resulting in a positive entropy change. The Gibbs free energy change must have a negative value for a reaction to occur [23]. A higher positive value for the enthalpy of dehydration means the temperature must increase for a more hydrophilic monomer to undergo a phase change (Equation (1)).

(1) $$\Delta G=\Delta H_{DH}-T\Delta S_{DH}$$

where Δ*G* is the Gibbs free energy change, Δ*H_DH_* is the enthalpy change of dehydration, *T* is the temperature, and Δ*S_DH_* is the entropy change of dehydration.

The desired phase change temperature is 40–43 °C in the nanoparticle complex. However, as the SPION core of the complex is hydrophobic, attachment reduces the LCST of the overall complex. The ratio of acrylamide in the copolymer when bound to a nanoparticle needs to be greater than that of the free copolymer to achieve the same LCST.

**Figure 2:**
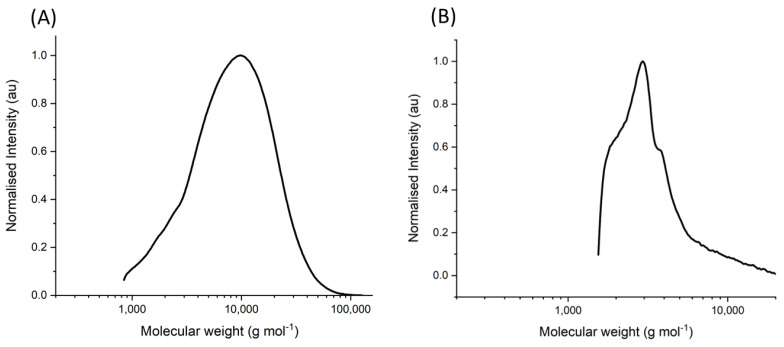
Gel permeation chromatogram of the synthesized copolymer. (**A**) The molecular weight distribution of NIPAM–acrylamide–methacrolein copolymer synthesized in free solution at a starting molar ratio of 45:45:10, respectively. (**B**) The molecular weight distribution of the equivalent copolymer synthesized on the surface of SPIONs at a starting molar ratio of 45:45:10, respectively. Molecular weight intensity is normalized to the logarithm of the peak maxima intensities.

Gel permeation chromatography was used to determine the extent of polymerization for the designed copolymer. A weight average molecular weight of 10,948 g mol^−1^ was determined, with a polydispersity (PDI) index of 2.01 (Figure 2A). Using the weight average molecular weight as the chain mass and assigning the molecular weight occupied by each monomer proportionally to the reactant molar ratio, the number of repeat units of each monomer was calculated. The number of NIPAM repeat monomers in this synthesized copolymer was 40 repeat units, acrylamide was 56 repeat units, and methacrolein was 14 repeat units. Using the assumption that the starting reaction molar ratio is equivalent to the composition in the final product is not an accurate representation of the true nature of the polymer and so must be observed with that in mind.

### 3.2. Nanoparticle Complex Assembly and Drug Release

**Figure 3:**
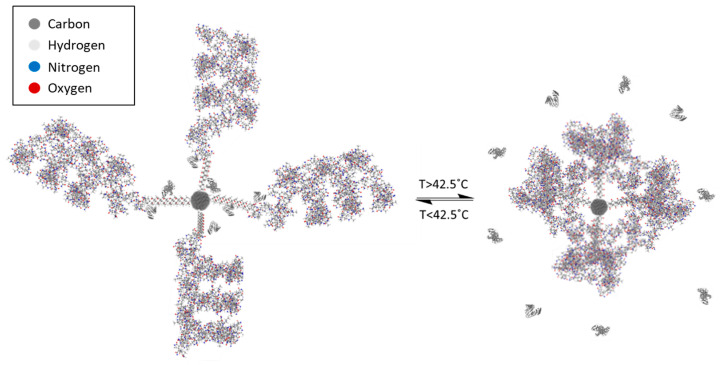
Release mechanism of the NCC upon radio wave stimulation. Schematic diagram illustrating the physiological mechanism of protein-bearing drug release from the NCC upon reaching the LCST. A visualization of the activation of the complex upon heating and the collapse of grafted polymer chains.

The nanoparticle–copolymer complex (NCC; Figure 3) was designed to perform as an activatable carrying moiety for a variety of chemotherapeutic agents. Varying the ratio of monomers in the designed copolymer yields a SPION-bound polymer capable of undergoing a phase transition at the desired temperature (Supplementary Figure S9). To assemble the complex, the copolymer was synthesized on the surface of DDMAT-stabilized SPION in situ and bound to the core by the stabilizing (and RAFT chain transfer) agent. The methacrolein in this reaction was added initially without co-monomers and allowed to react for two weeks. NIPAM and acrylamide were subsequently added and allowed to react for a further 48 h. This was performed with the intention of creating distinct block regions within the copolymer. The assumed block structure is desired for use in the nanoparticle–copolymer complex structure where the hydrophobic methacrolein core is synthesized on the inner shell of the copolymer. This protects the immobilized drug-bearing proteins and ensures that the outer layer of the complex is hydrophilic and biocompatible. Bovine serum albumin is believed to be bound to the complex through the formation of a Schiff base linkage between aldehyde groups on the particle-bound polymer and amine groups on the protein. When the polymer chains undergo a phase change at the LCST, the constriction on the protein–polymer bond is strained, and the release of the protein is stimulated (Figure 3).

**Figure 4:**
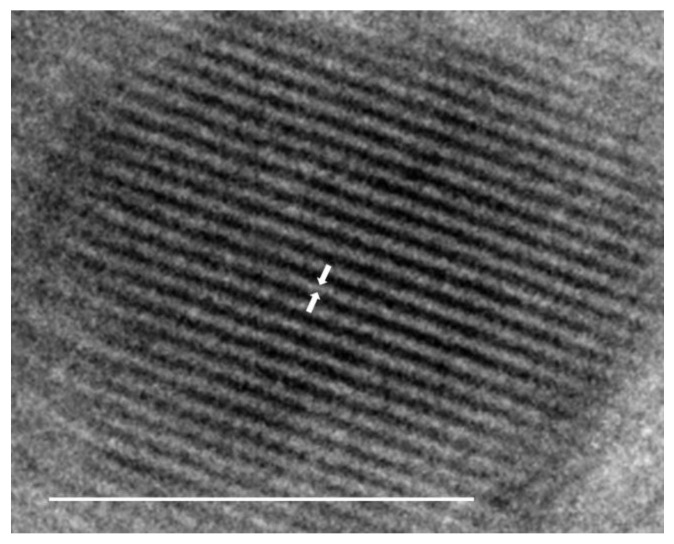
TEM image of spherical SPION core indicating the unit cell spacing. The scale bar is 5 nm, and the arrows indicate a spacing of 4.1 angstroms.

**Figure 5:**
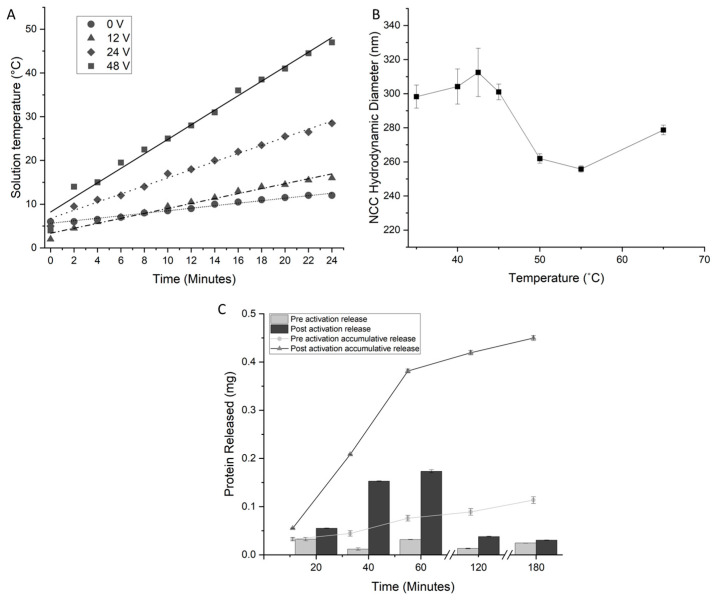
Properties of the NCC. (**A**) The induction heating rates of the NCC at a concentration of 100 mg/mL observed at differing AC circuit voltages from 0–48 V. (**B**) The temperature-dependent hydrodynamic diameter of NCC, as measured by dynamic light scattering. Swelling of the complex is observed until an LCST of 42.5 °C is reached, at which the hydrodynamic diameter decreases. (**C**) The release profile of BSA from protein-loaded NCC. Stimulated and passive releases are shown, as well as cycled and accumulative releases. The protein content was measured by a Bradford protein assay.

Figure 4 shows TEM images of the SPION core within the polymer complex; the single crystalline nature of the SPION core is visible, showing an average diameter of 7 nm (SD +/−1.8 nm). The unit cell spacing of the inverse spinel structure of magnetite is visible. Magnetic susceptibility measurements confirmed the superparamagnetic behavior of the SPION core with a closed M-H curve (Supplementary Figure S8). Dynamic light scattering data confirmed the hydrodynamic diameter of the nanoparticle–copolymer complex at 297 ± 42 nm at 37 °C (Figure 5B). The zeta potential data showed that the complex had a charge of −4.12 ± 4.7 mV (Supplementary Figure S10). The FT-IR spectra of the assembled complex showed data consistent with the free polymer, indicating no difference in the copolymer formed in the free solution or on the surface of SPIONs (Supplementary Figure S11). NMR data run on copolymers removed from the complex showed identical peaks to that of the free polymer (Supplementary Figure S12). The stability of the nanoparticle copolymer complex was tested over 90 days while stirring at 37.5 °C in PBS. There was no deviation in the hydrodynamic diameter over this time period, indicating the stability of the complex in physiological conditions. Although this does not take into account many physiological factors that may contribute to the degradation of the metabolism of the complex, it is an indication of the potential for stability in vivo [24].

Gel permeation chromatography was again used to determine the extent of polymerization for the copolymer on the surface of SPIONs (Figure 2B). After removal from the nanoparticle copolymer complex by ligand exchange, the samples were analyzed akin to the freely synthesized copolymer. A weight average molecular weight of 4502 g mol^−1^ was determined, and the PDI was found to be 1.41. The deliberate block synthesis method resulted in a reduced chain length in comparison to the freely synthesized copolymer due to the lower reactivity rate of the methacrolein monomer. Using the weight average molecular weight as the chain mass and assigning the molecular weight occupied by each monomer proportionally to the reactant molar ratio, the number of repeat units of each monomer was calculated. The number of NIPAM repeat monomers in this synthesized copolymer is 17 repeat units, acrylamide is 24 repeat units, and methacrolein is 6 repeat units.

The efficiency of the induction heating of the constructed NCC was assessed at varying input voltages from 0–48 volts. The observed temperature increases showed linear trajectories (Figure 5A) in line with the linear regime model [9]. As expected, higher input voltages resulted in a higher rate of heating for particles, proportional to the increase in voltage, e.g., a voltage of 48 volts showed a rate of +1.76 °C min^−1^. The heating rates were lower than that of SPIONs for the same concentration by mass per volume; this is a result of the copolymer chains occupying a higher proportion of the mass per volume of the particles than DDMAT occupies on SPIONs. Regardless, the NCC was heated sufficiently by induction heating above the activation temperature.

BSA was chosen as the carrier for chemotherapy agents because of its biocompatibility, availability, and the possibility of immobilization on polymers containing aldehyde functional groups [25]. This is a reversible condensation reaction between the aldehyde groups on the methacrolein component of the polymer and amine groups present on BSA [26]. Protein immobilization was performed analogously to the free copolymer; a total of 0.291 ± 0.027 mg of BSA was immobilized by 6 mg of the NCC. Approximately 4.85 ± 0.45% by the weight of NCC was assessed to be drug-carrying proteins after immobilization. This protein-carrying capacity is similar to that of the free polymer.

The LCST of the NCC was assessed by measuring the hydrodynamic diameter because of the coil-to-globular transition (Figure 5B). As the temperature increases, the complex swells due to the increased expansion of water within the structure with increasing temperature. Above 42.5 °C, a reduction in diameter is observed, indicating that the phase transition has occurred.

Paclitaxel was bound to BSA through association hydrogen bonding at a ratio of 1.1 drug molecules to every protein molecule, as shown by HPLC data (Supplementary Figure S13). Vinblastine was shown to bind to BSA at a molar ratio of 5.1:1 (Supplementary Figure S14). When compared to the literature results for the same experiment with human serum albumin (HSA), the loading ratio seen here is comparable [26,27].

Figure 5C shows the release profile of BSA from NCC over 7 days at physiological conditions. The active release consisted of inductively heating the NCC solution above and then cooling below the LCST of the complex. The first five cycles (over five days) show significant protein release with activation and negligible release with no activation. After five cycles, the amount of released protein drops off considerably, and after six cycles, no protein release can be detected. The passive release of proteins was a small fraction of the amount released by stimulation (Figure 5C). Protein release from the complex was observed when stimulated by radio waves. Protein release can be staggered over a number of days, and without stimulation, protein release is minimized (Figure 5C). This evidence of protein retention without heating serves as the basis for the potential of selective drug release from copolymers bound to SPIONs and thus allows for the “activation” of drug release. The protein release from the NCC was staggered over more cycles of the LCST than the free copolymer. This could be an effect of the close proximity of copolymer chains to each other, trapping protein molecules that would otherwise be released.

**Figure 6:**
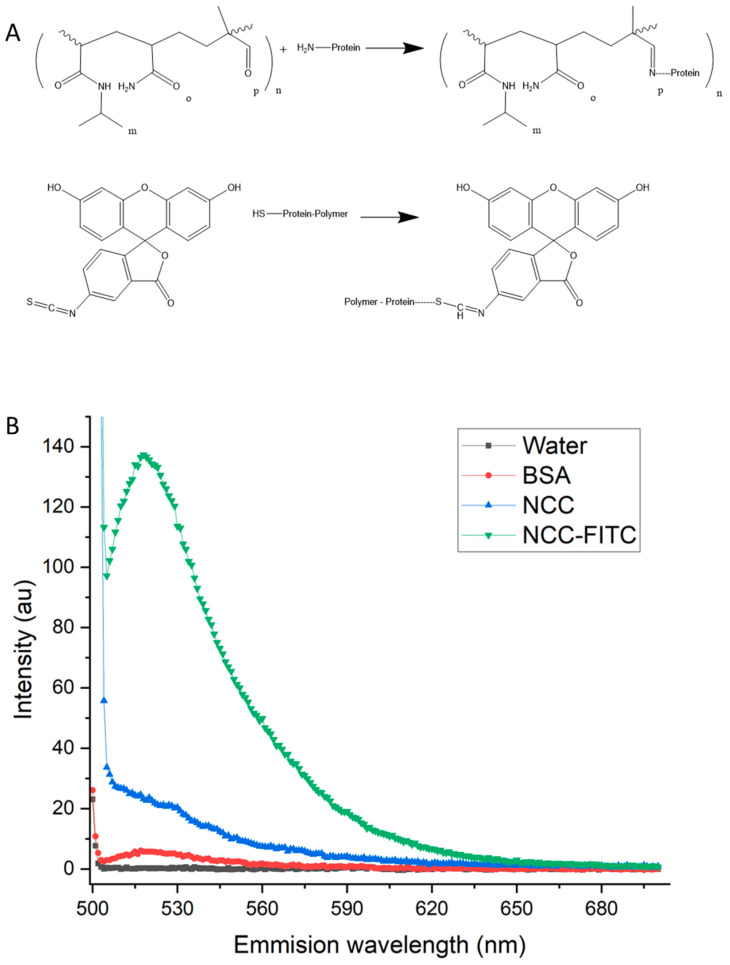
Mechanism and result of FITC binding to the NCC. (**A**) The reaction scheme detailing protein binding to the copolymer present on the NCC and the subsequent attachment of the fluorophore FITC to the bound protein. (**B**) The NCC-exhibiting fluorescence signal following binding with FITC. Fluorescence spectrum from 500 to 650 nm of water, 1 mg/mL solution of BSA, NCC, and NCC-FITC. The excitation wavelength of the sample was 495 nm.

Fluorophore attachment to BSA was achieved through FITC binding to sulfide groups and available amine groups (Figure 6A) following protein immobilization on the nanoparticle–polymer complex. The presence of FITC in the nanoparticle complex (FITC-NCC) was confirmed by visualization under UV light and fluorescence spectroscopy (Figure 6B).

The samples and controls were excited with a 495 nm-wavelength light from a Xenon flash lamp, and the emission spectrum was measured between 500 nm and 700 nm. Water and BSA alone showed negligible fluorescence. The NCC control showed some scattering-related signals, whereas the fluorescently tagged complex showed a clear and definite emission peak at 520 nm, i.e., FITC emission. The fluorophore attachment enables tracking in vitro but will also be important for clinical use in order to assess magnetic localization in vivo or to monitor tumor physiology during treatment. It is also possible to bind a fluorophore directly to the NCC. Again, this could be exploited to monitor nanoparticle movement, in contrast to protein and drug tracking.

### 3.3. Cell Viability Experiments with the Activatable Complex

**Figure 7:**
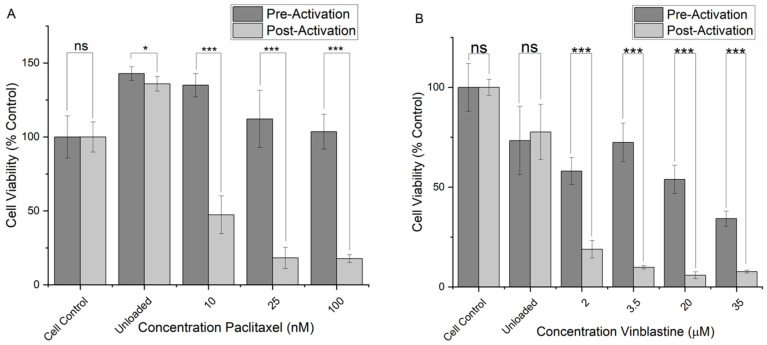
Cell viability was assessed by delivering chemotherapy agents to cells in vitro. The NCC was used to deliver paclitaxel and vinblastine to (**A**) RD cells, and (**B**) U-87 MG cells; the x-axis represents concentrations of drugs carried and released by the NCC. Cells refer to the cell-only control and are set to 100%. Unloaded refers to cells incubated with a 2 mg/mL solution of the NCC without bound drugs. All data are scaled as a percentage of cell survival normalized to the cell-only control. Significance was tested using a two-tailed *t*-test compared between pre- and post-activation for each concentration. P-values assessed between pre- and post-activation are represented as follows *** = *p* < 0.01, * = 0.5 < *p* > 0.05, ns = *p* > 0.5. Bars represent mean +/− standard deviation, n = 3.

The NCC was loaded with paclitaxel (NCC-PAC) or vinblastine (NCC-VIN) and tested for activity against rhabdomyosarcoma (RD) and glioblastoma (U-87 MG) cell lines, respectively. Paclitaxel was delivered to RD cells to act as an initial model drug, vinblastine was delivered to U-87 MG cells as a second model drug due to the U-87 MG cell’s resistance to paclitaxel. FACS analysis was used to determine the degree of cell death after incubation with the complex and before and after activation (Figure 7). An increase in cell viability was seen when RD cells were subjected to a 2 mg/mL concentration of unloaded NCC; this was likely due to the availability of iron as a micronutrient allowing RD cells to proliferate [28]. In the RD cells, neither pre-activation-unloaded NCC nor pre-activation NCC-PAC caused cell death at any concentrations tested (up to 10 nM; Figure 7A). The incubation of the unloaded NCC with U-87 MG cells did show limited cell death, pre- and post-activation. This is likely due to the U-87 MG cell’s susceptibility to high concentrations of iron present in the solution, showing a much lower tolerance than RD cells [29]. However, the incubation of the NCC-VIN with U-87 MG cells did show some degree of cell death from pre-activation particles (Figure 7B).

Activation was achieved by placing the cell culture plate within an induction coil for a predetermined time, dependent on nanoparticle concentration, to raise the temperature to 43 °C. After activation with AMF, a high degree of cell death was seen in both cell lines. In RD cells, 25 nM NCC-PAC resulted in 79 ± 6% cell death when compared to control cells after activation (Figure 7A). At the same concentration, there was no pre-activation cell death seen (Figure 7A, grey bars). Near-total cell death (95 ± 4%) was seen in U-87 MG cells after incubation with NCC-VIN equivalent to 5 μM of vinblastine (Figure 7B, black bars). Thus, even though there is some pre-activation cell death, there is still a high degree of discrimination between pre- and post-activation for the NCC construct (*p* < 0.01, Figure 7B). The fact that unloaded NCC does not cause significant cell death after AMF activation indicates that there is minimal direct hyperthermic damage to the cells and that the temperature increase in the particles serves only to modify the polymer to cause drug release. Throughout the cell lines tested, there was no significant difference in cell death observed for activation vs. pre-activation unloaded concentrations of NCC. This indicates that there is no cell death caused by the activation through the application of radio waves, reinforcing the hypothesis that the stimulus method is biocompatible. When drugs were delivered with activated NCC, cell death was significantly higher than the same concentration with the NCC left in a “pre-activation state”. Particularly in low concentrations of the delivered drugs, significant cell death was caused by the activated NCC, but little or no significant cell death was observed for the pre-activation sample. This indicates a high degree of selective therapeutic effect upon the radio wave activation of the complex.

**Figure 8:**
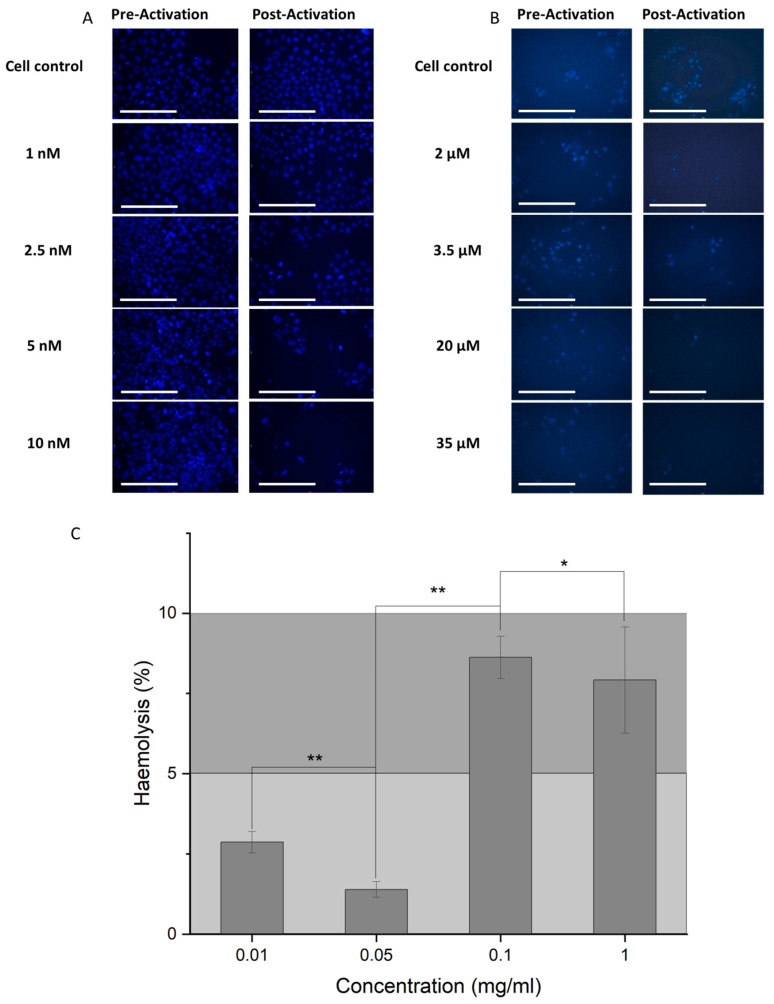
Visual demonstration of selective cell death by the stimulation of the NCC by radio waves. (**A**) RD cells and (**B**) U-87MG cells imaged under 495 nm light when stained with Hoechst, after treatment with pre-activation NCC (left), and post-activation NCC (right) at the following concentrations listed vertically downwards; (**A**) cell control, 1 nM, 2.5 nM, 5 nM, and 10 nM, and (**B**) cell control, 2 μM, 3.5 μM, 20 μM, and 35 μM. The scale bar is equal to 100 μm. (**C**) The hemolysis percentages highlighting the biocompatibility of the unloaded NCC. PBS was used as a negative control and water as a positive control to indicate hemolysis. Negligible hemolysis is observed for concentrations from 0.01 to 1 mg/mL ION–polymer. *p*-values assessed are represented as follows ** = 0.05 < *p* > 0.01, * = 0.5 < *p* > 0.05. Bars represent mean +/− standard deviation, n = 3.

As a further test to demonstrate cell death post-activation, cells were imaged by microscopy after Hoechst staining. Fluorescent images show a decrease in the number of adherent cells in the presence of the activated loaded nanoparticle complex for both NCC-PAC (Figure 8A) and NCC-VIN (Figure 8B).

In order to demonstrate biocompatibility in vivo, the NCC was incubated with blood, and the degree of hemolysis was determined (Figure 8C). At the highest concentration of 1 mg/mL NCC, hemolysis was seen to be 7.9 ± 1.6%. Compounds are assumed to be generally biocompatible if hemolysis is below 10% [30].

**Figure 9:**
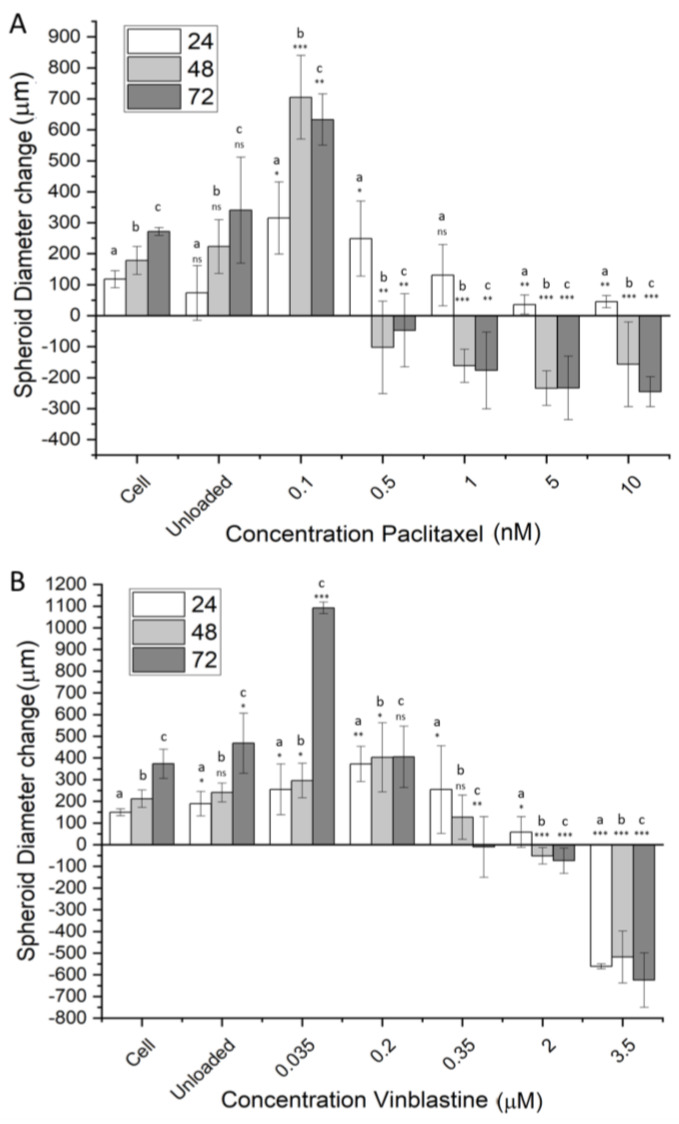
The effect of drug delivery by the NCC on spheroid models. Spheroid diameter changes from t = 0 in the presence/absence of varying concentrations of activated NCC–drug. (**A**) shows RD spheroid diameter change when paclitaxel is delivered by the NCC, and (**B**) shows the diameter change for U87-MG cells with the delivery of vinblastine. The change in diameter is shown over a period of 72 h. Diameters were measured using three dimensions of the radius and were performed in triplicate. Cells refer to the cell-only control and are set to 100%. All data are listed as spheroid diameter change in comparison to the diameter at t = 0. Significance was tested using a two-tailed *t*-test that compared between the sample and the cell control for that time point; a represents a comparison to 24 h, b to 48 h, and c to 72 h. *p*-values assessed are represented as follows *** = *p* < 0.01, ** = 0.05 < *p* > 0.01, * = 0.5 < *p* > 0.05, ns = *p* > 0.5. Bars represent mean +/− standard deviation, n = 3.

In addition to 2D cell culture models, 3D spheroid models were used as a representation of avascular tumors. Figure 9A shows the diameter change in RD spheroids from the addition of activated NCC-PAC. Change in diameter is shown over a period of up to 72 h. It is clear from Figure 9A that a greater reduction in spheroid diameter is seen with increasing concentrations of NCC-PAC. After 24 h, there was no significant deviation in spheroid diameter from the control up to 5 nM concentration. Concentrations greater than this showed a much smaller diameter increase than that of the cell control (*p* < 0.05, Figure 9A). After 48 h, a large increase in spheroid diameter was observed for 0.1 nM drug concentration. Drug concentrations of 0.5 nM and greater showed a significant decrease in spheroid diameter after 48h (Figure 9A). After 72 h, spheroid diameter reduced after incubation with 0.01 nM drug, but the diameter increase was still significantly greater than that of the control. After 72 h, concentrations of 0.5 nM and above showed a diameter reduction in comparison to the cell. Additionally, for higher concentrations, after 72 h of incubation, some of the spheroids started to disintegrate entirely, contributing to larger deviations in the measured diameter.

Using the same method, the capacity of the NCC to deliver vinblastine to U-87 MG cells was investigated (Figure 9B). There was no difference in spheroid diameter change observed for unloaded particles in comparison to the cell control over the entirety of the time observed (Figure 9B). After 24 h, no significant difference was observed for diameter change in comparison to the control up to 2 μM of the added drug. At 3.5 μM of the added drug, there was a dramatic reduction in spheroid diameter (Figure 9B), indicating a critical concentration of vinblastine delivered. Briefly, 48 h after incubation, a concentration of 2 μM of the drug showed a significant diameter reduction (*p* < 0.05), and spheroids subject to a concentration of 3.5 μM drug started to lose integrity. After 72 h, incubation concentrations of 350 nM and greater showed significant reductions in cell viability.

The spheroid models showed good biocompatibility for the unloaded NCC, as no significant cell death was seen with any cell line. The concentrations of the drugs delivered showed a reduction in spheroid diameter compared to the untreated control, exhibiting a level of penetration into the structure. Generally, spheroid diameter continued to reduce as incubation time increased. Higher concentrations of delivered drugs generally caused a quicker reduction in spheroid diameter.

**Figure 10:**
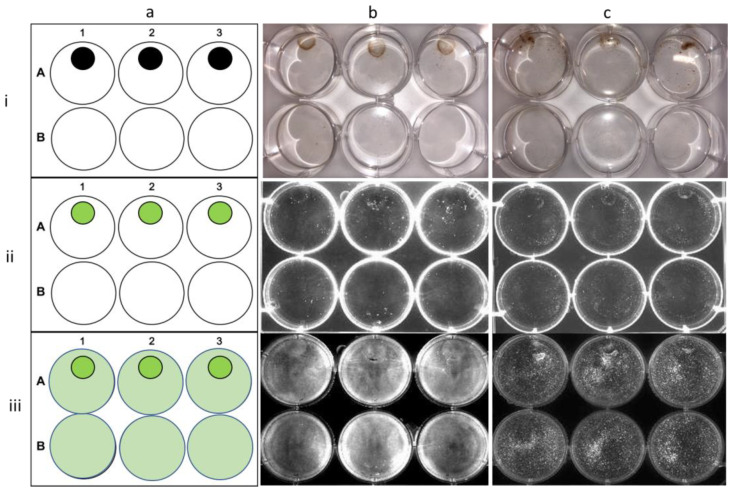
Magnetic localization of the NCC within cells. Demonstration of the magnetic direction ability of NCC in localizing RD cells (column **b**) and U-87 MG cells (column **c**) around a permanent magnetic field. (Column **a**) shows the position of permanent magnets in the plate and the regions of fluorescence. Permanent magnets were placed in wells A1, A2, A3, and B2. NCC-FITC-bearing cells were mixed with control cells in wells A1, A2, A3, and B1. Well B3 contained no NCC-bearing cells and no magnetic field as a control. Row (**i**) depicts the visible light image of the plate. Row (**ii**) displays the same plate fluorescently imaged at an excitation wavelength of 495 nm. Row (**iii**) shows the plate imaged fluorescently as in (**ii**) but with cell membrane dye present.

Cells (RD; Figure 10b, and U-87 MG; Figure 10c) containing the NCC were regionally fixed as a demonstration of the magnetic directable ability. Permanent magnets were placed in wells A1, A2, A3, and B2, and NCC-containing cells were mixed with non-NCC-containing cells and allowed to settle for 24 h. NCC-containing cells were mixed with non-NCC-containing cells in wells A1, A2, A3, and B1, whereas B3 contained no NCC-containing cells and no magnetic field as a control. Cells with internalized NCC particles are directed to settle under the permanent magnetic field when applied; this is exhibited by the presence of FITC fluorescence in these cells. Furthermore, the magnetic field did not affect the distribution of non-NCC-containing cells (membrane stained) within the plate, nor the cell viability of either population.

## 4. Conclusions

We have reported here the first development of a highly discriminant, directable, and controllable method of chemotherapy drug delivery. Through a variety of interdisciplinary techniques, a novel superparamagnetic iron oxide nanoparticle–poly–(methacrolein-b-(NIPAM-co-acrylamide)) complex has been successfully synthesized and analyzed. The grafted polymer has been meticulously engineered to undergo a phase transition at the critical temperature range of 41–43 °C. We have shown that the complex is capable of immobilizing and releasing, upon radio wave activation, drug-carrying bovine serum albumin. We have shown that the complex, once drug-loaded and activated, can induce cell death by the controlled release of paclitaxel and vinblastine. The complex can also be conjugated with a fluorophore and can be easily manipulated by magnetic fields to gather in specific locations for treatment. Consequently, this complex provides a method for chemotherapy agent delivery that has the potential to provide effective treatment while eliminating side effects.

## 5. Patents

The design of the nanoparticle–copolymer complex and its use as an activatable drug delivery platform, including the design of the novel copolymer and the activation method, are covered in the following patent application. The authors of this manuscript are the same as the inventors listed in the patent.

Applicants: Oxford University Innovation Limited. Inventors; White, Benjamin. Townley, Helen Elizabeth. International Application No: PCT/GB2021/052104. Status of application: Patent Pending.

## Data Availability

Not applicable.

## Supplementary Materials

The following supporting information can be downloaded at: https://www.mdpi.com/article/10.3390/ma16062482/s1, Figure S1: Unbound PNIPAM–acrylamide–methacrolein visualized. Figure S2: FT-IR of unbound PNIPAM–acrylamide–methacrolein copolymer. Figure S3: NMR spectra of unbound PNIPAM–Acrylamide-Methacrolein copolymer. Figure S4: The effect of the change in a molar ratio of constituent monomers on the LCST of the free copolymer. Figure S5: Protein immobilized on unbound PNIPAM–acrylamide–methacrolein (45-45-10) copolymer. Figure S6: NCC after synthetization. Figure S7: NCC (Copolymer ratio PNIPAM–acrylamide–methacrolein 45:45:10) below and above the LCST. Figure S8: Magnetic susceptibility data of NCC, at 100 and 300 K. Figure S9: The effect of the change in a molar ratio of constituent monomers on the LCST of NCC. Figure S10: Zeta potential of NCC. Figure S11: FT-IR of synthesized PNIPAM, acrylamide, and methacrolein polymer on the surface of SPIONs. Figure S12: H1 NMR (top) and C13NMR (bottom) spectrums of bound copolymer. Figure S13: LC chromatogram demonstrating the Paclitaxel immobilized on BSA. Figure S14: LC chromatogram demonstrating the Vinblastine immobilized on BSA. Figure S15: TEM images of the SPION core of the NCC before (A) and after (B) RAFT polymerization on the SPION surface.
