# Supplementary material for: Radio Wave-Activated Chemotherapy—A Novel Nanoparticle Thermoresponsive Copolymer Drug Delivery Platform
Source: Materials (Basel). 2023 Mar 21;16(6):2482. doi: 10.3390/ma16062482 (PMC10059094; doi:10.3390/ma16062482)
Supplement: Supplementary file 1 [file materials-16-02482-s001.zip › materials-2106039-supplementary.pdf]

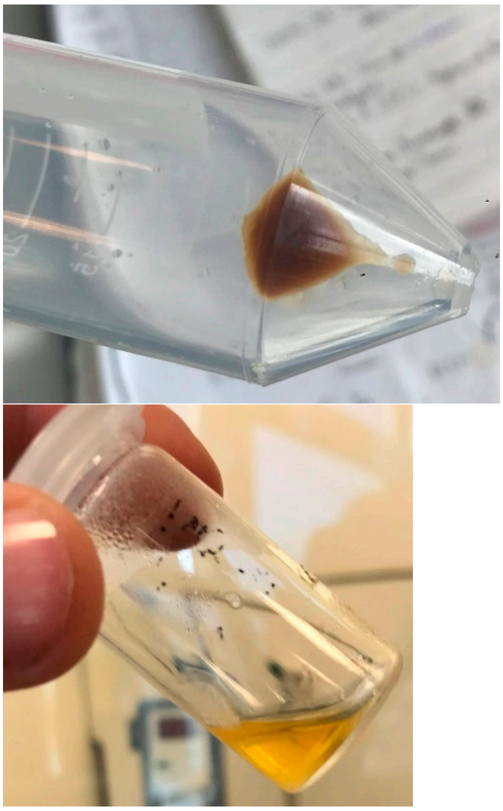

Supplementary Figure S1: Unbound PNIPAM-Acrylamide-Methacrolein (40:50:10) copolymer pellet after centrifugation following synthesis (left) and 10 mg copolymer after being dissolved in 1 ml water (right).

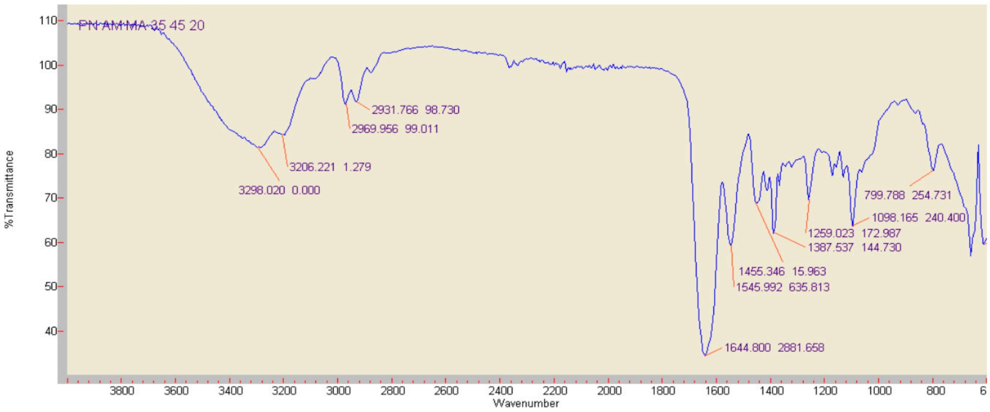

Supplementary Figure S2:FT-IR of unbound PNIPAM-Acrylamide-Methacrolein copolymer after washing and subsequent freeze drying.

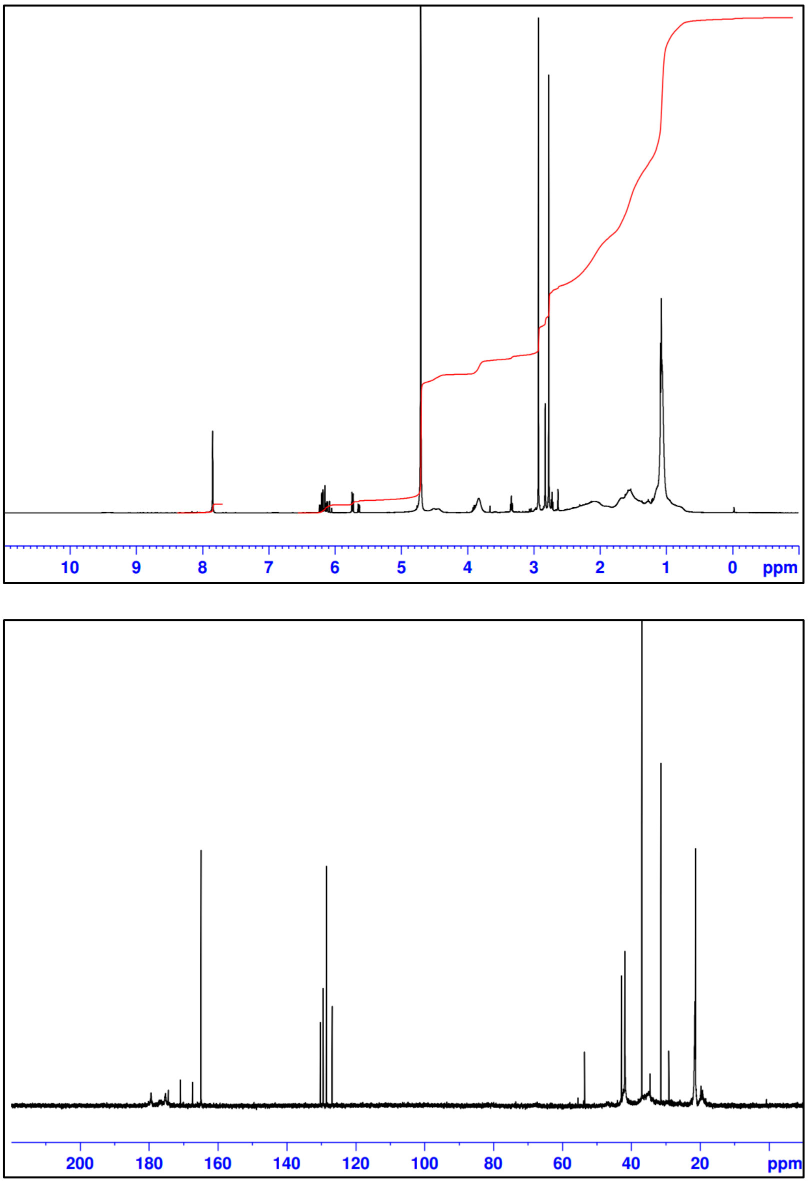

Supplementary Figure S3:NMR spectra of unbound PNIPAM-Acrylamide-Methacrolein copolymer H1 NMR top, and C13 NMR bottom.

Supplementary Figure S4: The effect of the change in molar ratio of constituent monomers on the LCST of the free copolymer

N-isopropylacrylamide	Acrylamide	Methacrolein	LCST/Release temp (˚C)
50	30	20	27
40	40	20	36
35	45	20	43
35	50	15	48

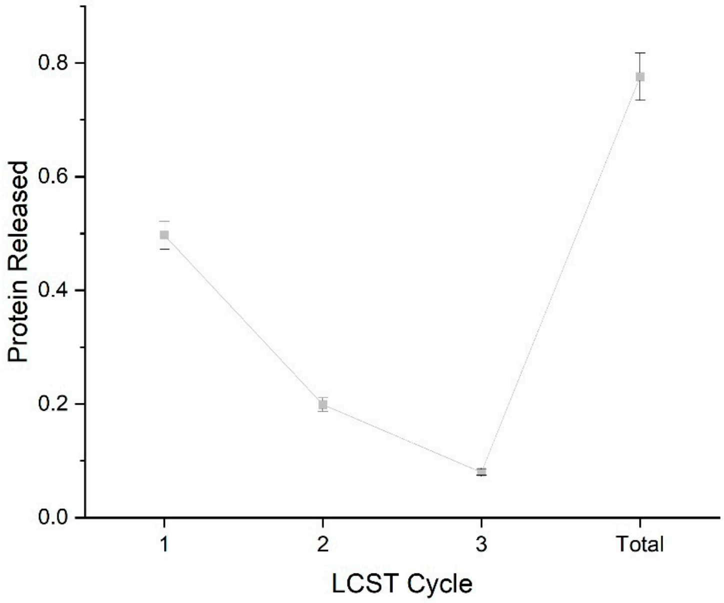

Supplementary Figure S5:Protein immobilised on unbound PNIPAM-Acrylamide-Methacrolein (45-45-10) copolymer. A total of 0.77 mg of BSA was immobilised by 8 mg of polymer. Around 9% by weight of polymer can be drug carrying proteins.

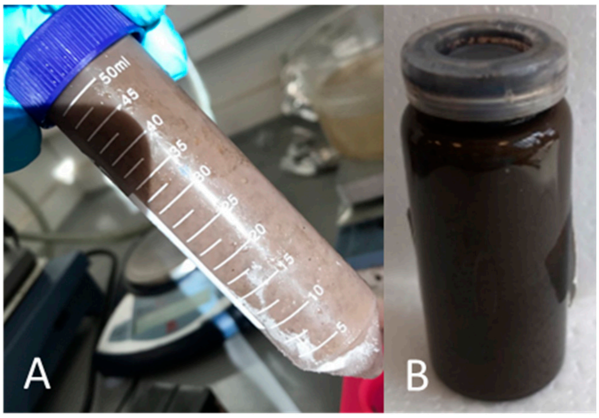

Supplementary Figure S6:NCC after precipitation in cold Diethyl-ether (A) and in a 100mg/ml solution in water (B).When precipi-tated the NCC takes on a cloudy dark brown/grey colour. When soluble the NCC takes on a dark brown/black colour.

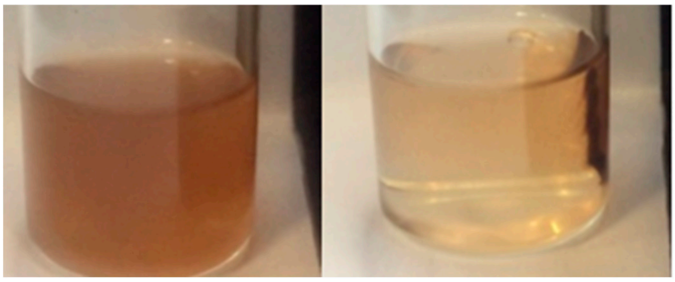

Supplementary Figure S7: NCC (Copolymer ratio PNIPAM-Acrylamide-Methacrolein 45:45:10) below (left) and above (right) the lower critical phase transition temperature of the complex. A neodymium magnet is placed to the right of each vial and exerts a permanent magnetic field attracting particles to the right hand side of the vial.

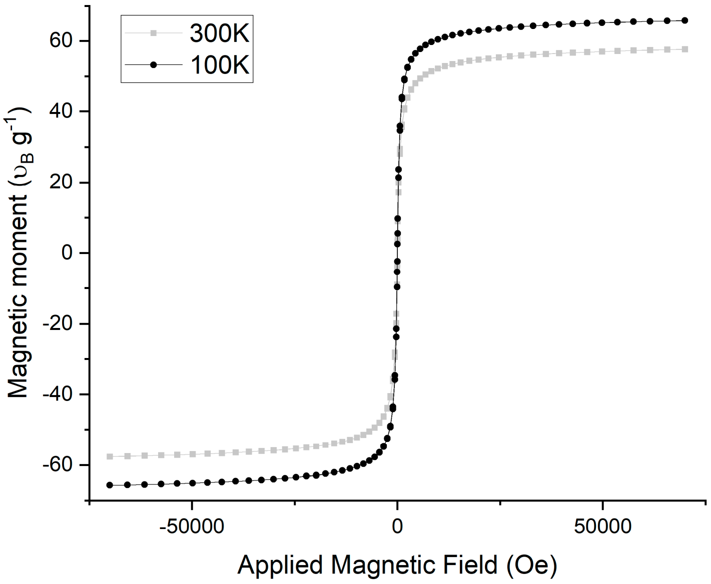

Supplementary Figure S8:Magnetic susceptibility data of NCC, at 100 and 300K

N-isopropylacrylamide	Acrylamide	Methacrolein	LCST/Release temp (˚C)
50	30	20	27
40	40	20	36
35	45	20	48
45	45	10	40-42

Supplementary Figure S9:The effect of the change in molar ratio of constituent monomers on the LCST of NCC

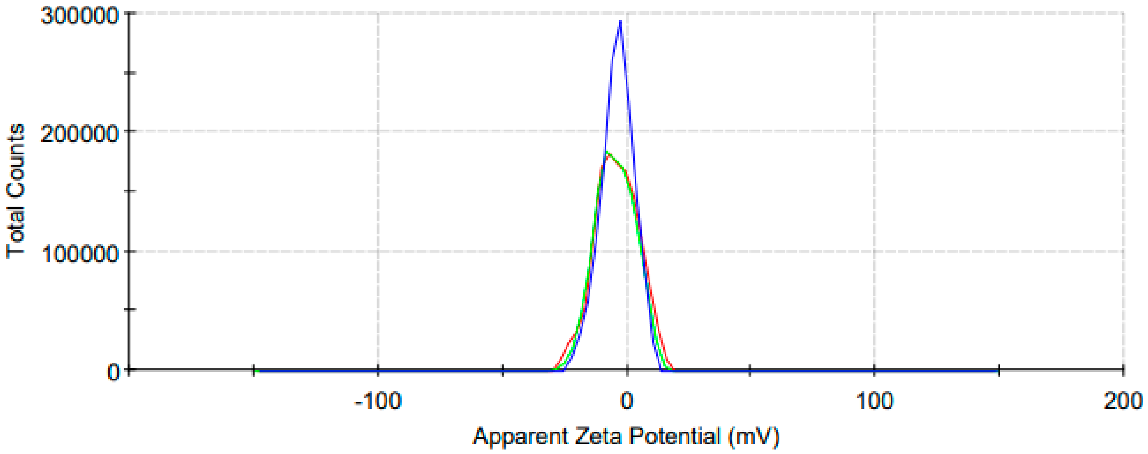

Supplementary Figure S10:Zeta potential of NCC over three repeat measurements, an average zeta potential of -4.12mV was observed.

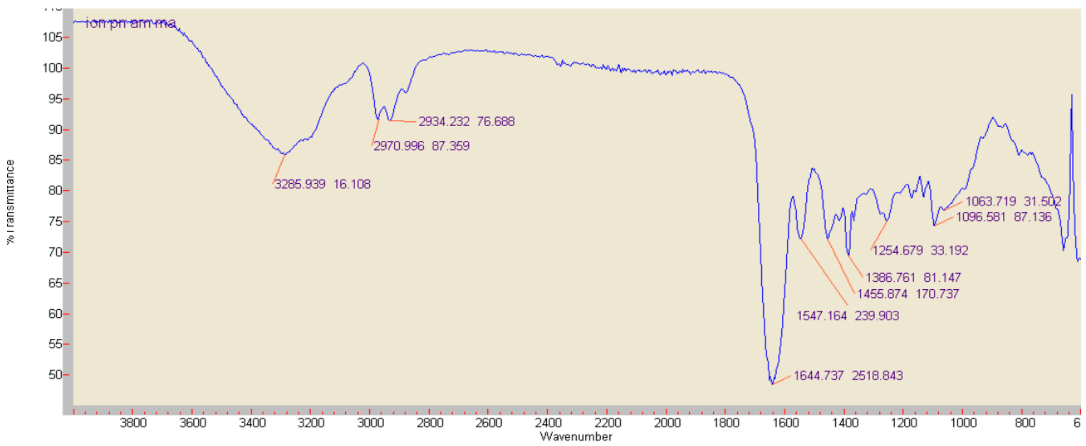

Supplementary Figure S11:FT-IR of synthesised PNIPAM, acrylamide and methacrolein polymer on the surface of SPIONs in a molar ratio of 45:45:10. Showing an identical FT-IR spectrum to that of the unbound copolymer.

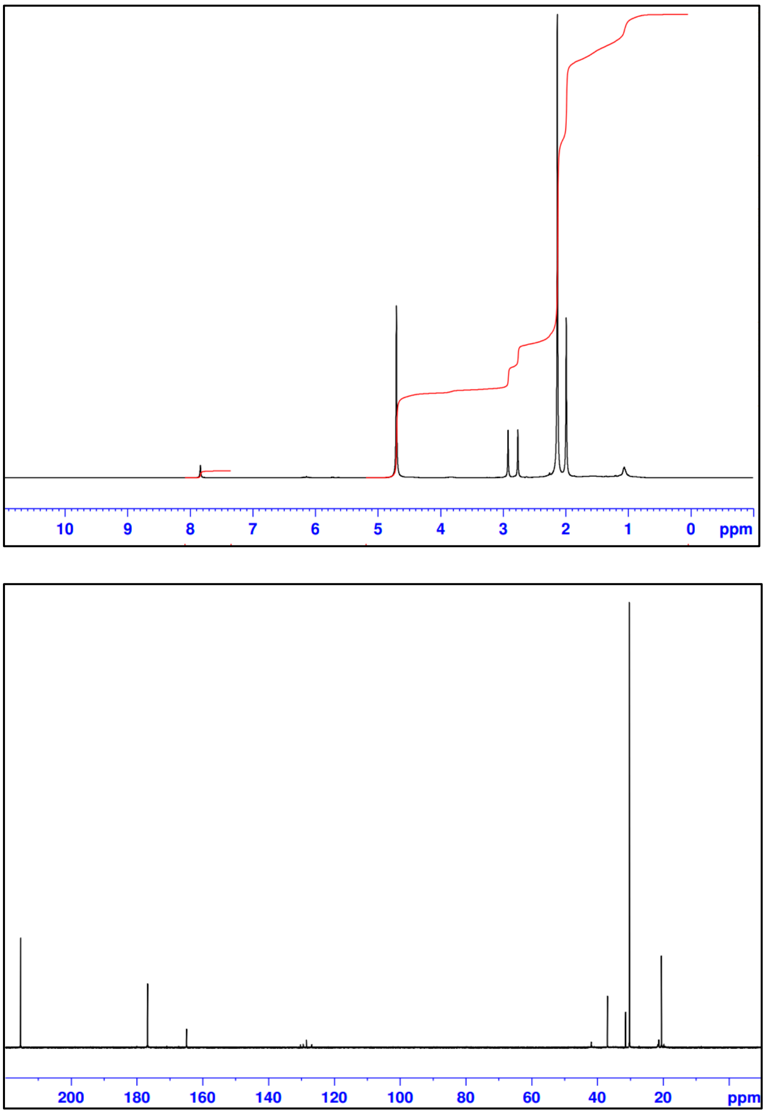

Supplementary Figure S12:H1 NMR (top) and C13NMR (bottom) spectrums of bound copolymer, following removal by ligand exchange. Confirming the desired copolymer identity and showing a similar spectrum to that of the unbound copolymer.

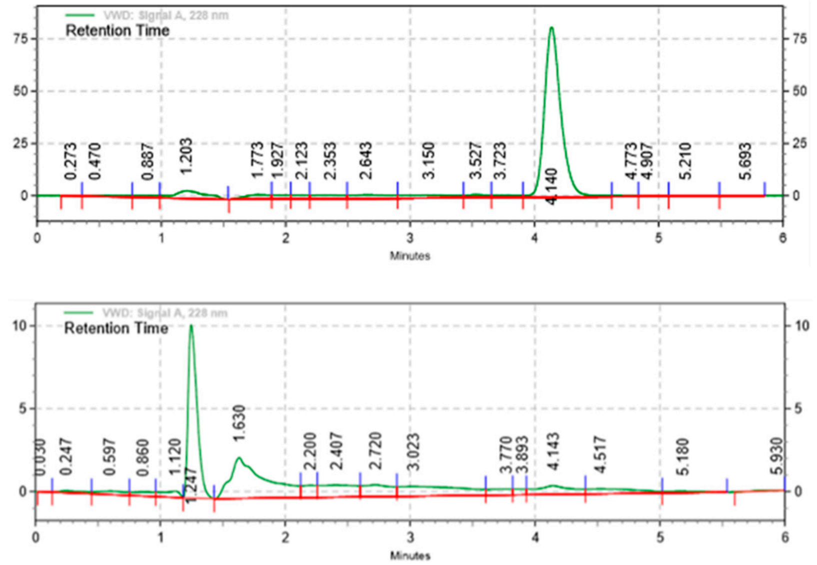

Supplementary Figure S13: LC chromatogram demonstrating the Paclitaxel peak present at 4.14, a known concentration of 600 µM (top) and a signal from a 0.1mg/ml BSA-PAC complex (bottom). *BSA degradation products are present from 1.1-1.65 minutes and the paclitaxel peak can be observed at 4.14 minutes.*

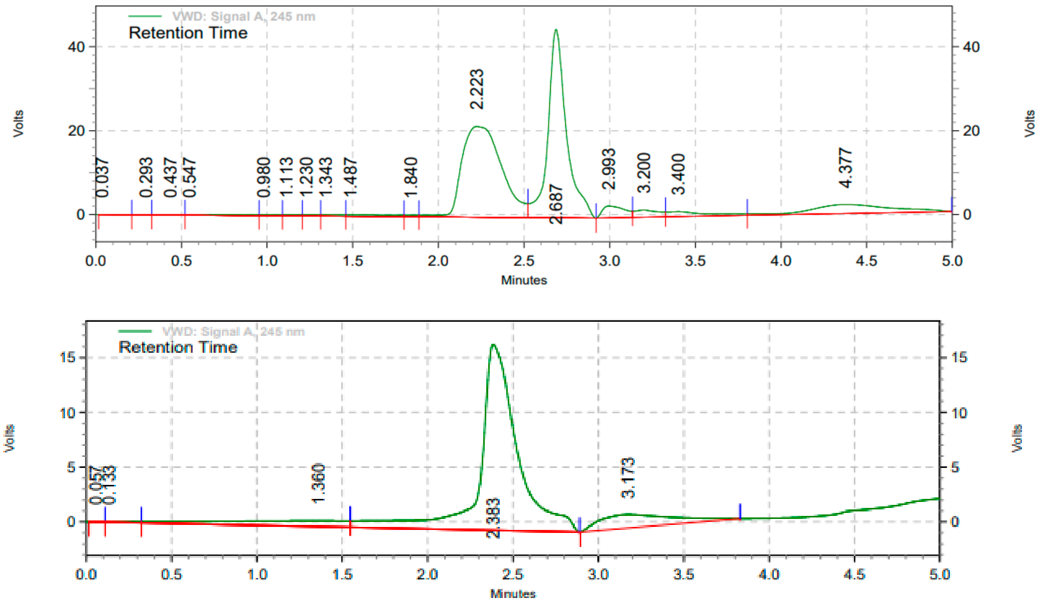

Supplementary Figure S14: LC chromatogram demonstrating the vinblastine peak present at 2.2—2.3 minutes of a known concentration of 600 M (top) and a signal from a 0.1 mg/ml BSA-VIN complex after BSA has been degraded in trypsin(bottom).

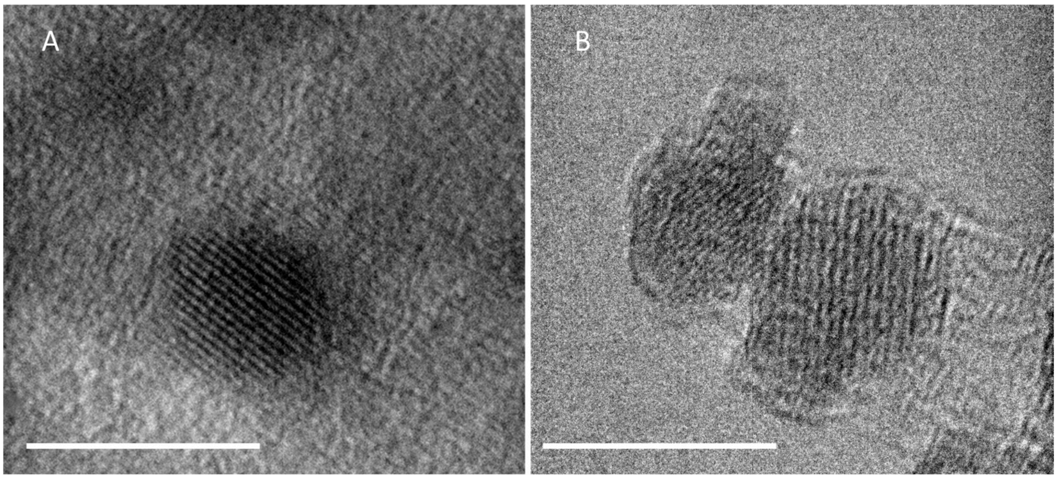

Supplementary Figure S15:TEM images of the SPION core of the NCC before (A) and after (B) RAFT polymerization on the SPION surface. Each scale bar represents 5 nm.
